# Basonuclin Regulates a Subset of Ribosomal RNA Genes in HaCaT Cells

**DOI:** 10.1371/journal.pone.0000902

**Published:** 2007-09-19

**Authors:** Shengliang Zhang, Junwen Wang, Hung Tseng

**Affiliations:** 1 Department of Dermatology, University of Pennsylvania, Philadelphia, Pennsylvania, United States of America; 2 Center for Bioinformatics, Department of Genetics, University of Pennsylvania, Philadelphia, Pennsylvania, United States of America; 3 Department of Computer and Information Science, University of Pennsylvania, Philadelphia, Pennsylvania, United States of America; 4 Cell and Developmental Biology, University of Pennsylvania, Philadelphia, Pennsylvania, United States of America; 5 Center for Research on Reproduction and Women's Health, University of Pennsylvania, Philadelphia, Pennsylvania, United States of America; Institute of Genetics and Molecular and Cellular Biology, France

## Abstract

Basonuclin (Bnc1), a cell-type-specific ribosomal RNA (rRNA) gene regulator, is expressed mainly in keratinocytes of stratified epithelium and gametogenic cells of testis and ovary. Previously, basonuclin was shown in vitro to interact with rRNA gene (rDNA) promoter at three highly conserved sites. Basonuclin's high affinity binding site overlaps with the binding site of a dedicated and ubiquitous Pol I transcription regulator, UBF, suggesting that their binding might interfere with each other if they bind to the same promoter. Knocking-down basonuclin in mouse oocytes eliminated approximately one quarter of RNA polymerase I (Pol I) transcription foci, without affecting the BrU incorporation of the remaining ones, suggesting that basonuclin might regulate a subset of rDNA. Here we show, via chromatin immunoprecipitation (ChIP), that basonuclin is associated with rDNA promoters in HaCaT cells, a spontaneously established human keratinocyte line. Immunoprecipitation data suggest that basonuclin is in a complex that also contains the subunits of Pol I (RPA194, RPA116), but not UBF. Knocking-down basonuclin in HaCaT cells partially impairs the association of RPA194 to rDNA promoter, but not that of UBF. Basonuclin-deficiency also reduces the amount of 47S pre-rRNA, but this effect can be seen only after cell-proliferation related rRNA synthesis has subsided at a higher cell density. DNA sequence of basonuclin-bound rDNA promoters shows single nucleotide polymorphisms (SNPs) that differ from those associated with UBF-bound promoters, suggesting that basonuclin and UBF interact with different subsets of promoters. In conclusion, our results demonstrate basonuclin's functional association with rDNA promoters and its interaction with Pol I in vivo. Our data also suggest that basonuclin-Pol I complex transcribes a subset of rDNA.

## Introduction

Basonuclin (Bnc1) is a zinc finger transcription factor expressed mainly in the keratinocytes of stratified epithelium and the reproductive germ cells of testis and ovary [1], [2]. Basonuclin is localized at the rDNA clusters on acrocentric chromosomes during mitosis [3], a typical behavior of Pol I-associated factors. Consistent with this localization, basonuclin's zinc fingers interact with rDNA promoter at three highly conserved binding sites [3]–[5]. No interaction was detected between basonuclin and beta-satellite DNA, which is a repetitive sequence also localized near the rDNA clusters on the short arms of human acrocentric chromosomes [3]. These results suggest that basonuclin's interaction with rDNA promoter is specific and not related to the repetitive nature of the rDNA array. Basonuclin stimulates transcription from a co-transfected rDNA promoter and basonuclin zinc fingers can act as a dominant-negative agent to inhibit Pol I transcription in oocytes [5]. Most interestingly, when basonuclin was knocked-down in mouse oocytes, the number of Pol I transcription foci were reduced, and the incorporation of BrU by the remaining foci was not affected [6]. This observation suggests that basonuclin regulates a subset of rDNA. Another intriguing issue is the relationship of basonuclin and the ubiquitous Pol I regulator UBF, which are co-localized on the same chromosomal loci in mitotic keratinocytes [3]. However, DNase I footprints of basonuclin and UBF overlap [3]–[5], which led to our question whether they interact with the same promoter molecule [3], [5].

Here we describe a study of basonuclin's interaction with rDNA promoters in the HaCaT cells, a spontaneous established human keratinocyte cell line [7]. To validate basonuclin's role in rRNA transcription, we also established a basonuclin knock-down model in the HaCaT cells. Our data lead us to propose some features of basonuclin's role in regulation of rRNA synthesis.

## Results

### Basonuclin interacted with rDNA promoter in HaCaT cells

**Figure 1:**
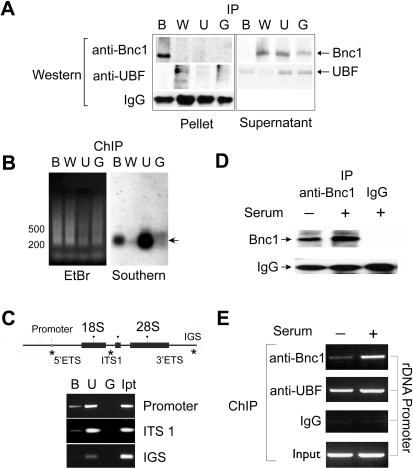
Basonuclin interacts with rDNA promoter in vivo. A, Western analyses of immunoprecipitated proteins from HaCaT cell lysate. The IP antibodies are shown above the Western blot; B, anti-basonuclin (alpha-hB34), U, anti-UBF, W, anti-WT protein, and G, IgG from a naive rabbit. The detecting antibodies are indicated on the left. The target proteins were monitored in both the pellet and supernatant. Note that no cross-reactivity was observed. B, The resolution of the ChIP assay was assessed by a Southern analysis. A primer was ligated to the immunoprecipitated DNA, which was then amplified by PCR. The amplified DNA was separated by agarose electrophoresis and visualized by ethidium bromide (EtBr). The DNA was then transferred to a nylon membrane and probed with an rDNA promoter probe (Southern). The chromatin immunoprecipitation (ChIP) antibodies are indicated above the gel by letters described in (A). A DNA size marker is shown on the left. The DNA fragments detected by the probe are indicated with an arrow on the right. C, Basonuclin's association with three regions of rDNA was investigated with ChIP-PCR. The top panel depicts a generic rDNA transcription unit. Indicated are the promoters, the rRNA coding sequences (18S and 28S), the external and internal transcribed spacers (ETS and ITS) as well as the intergenic spacer (IGS). Regions tested by PCR are indicated with a (*). Twice-ChIP-precipitated DNAs were used as template for PCR, whose products were analyzed by electrophoresis (lower panel). The ChIP antibodies are indicated above the gel (B, U, G, as in A, Ip, Input DNA). PCR specificity is shown on the right. D, Basonuclin level in HaCaT cells cultured in the presence (+) and absence (-) of serum. Basonuclin was immunoprecipitated from cell lysate and analyzed by Western blot. The precipitation antibodies are indicated above the gel image and the Western detecting antibodies on the left. E, The association of basonuclin and UBF to rDNA promoter in the presence and absence of serum. ChIP-precipitated DNA was used as templates for PCR detection of the rDNA promoter. ChIP-antibodies are listed to the left of gel image.

Previously, in vitro DNase I foot-printing detected highly-conserved basonuclin–binding sites within the human and mouse rDNA promoters [3]–[5]. To verify this result, ChIP assays were performed to examine basonuclin's association with rDNA promoter in HaCaT cells. HaCaT cells retained characteristics of proliferative basal keratinocytes [7], where basonuclin was highly expressed [8]. To precipitate basonuclin-associated chromatin, we used an affinity-purified anti-human-basonuclin antibody (alpha-hB34), which was raised against the full-length basonuclin and shown to detect basonuclin in Western blot as well as in immunocytochemistry [3]. The alpha-hB34 antibody precipitated from HaCaT cell extract a protein with molecular weight identical to that of basonuclin (i.e., 120k)(**Fig. 1A**) [3], [9], [10]. The amount of antibody used was sufficient to remove all soluble basonuclin (**Fig. 1A**), suggesting a quantitative precipitation. We used the up-stream binding factor (UBF), a ubiquitous Pol I transcription factor, as a positive control for interaction with rDNA promoter. The negative controls were Wilms' tumor protein (WT-1), a zinc finger transcription factor for Pol II [11], [12], and the normal rabbit IgG. To detect basonuclin's association with rDNA promoter, chromatin was twice precipitated by individual antibodies. The precipitated chromatin DNA was ligated to PCR primers and amplified. The amplified DNA was subjected to a southern analysis, which showed that only DNA cross-linked with basonuclin and UBF contained the rDNA promoter sequence (**Fig. 1B** **)**. This analysis provided the first evidence of basonuclin's association with rDNA promoter in vivo. The southern analysis also showed that basonuclin- and UBF-associated rDNA promoter fragments ranged between 0.2–0.4 kb (i.e., the resolution of the ChIP assay) (**Fig. 1B**). This fragment length was sufficient to resolve basonuclin/DNA interaction with various regions of the rDNA transcription unit. PCR primers were designed to examine three regions of rDNA, i.e., the promoter, the internally transcribed spacer (ITS) and the intergenic spacer (IGS). Most basonuclin appeared to associate with the promoter, much less with the ITS and none with the IGS (**Fig. 1C**). UBF, on the other hand, associated equally with the promoter and ITS, as shown previously [13].

To examine if basonuclin's association with rDNA promoter was regulated by extracellular signals, we performed ChIP assays with HaCaT cells cultured with or without serum for 24 h. Western analysis showed that precipitable basonuclin was reduced in the absence of serum (**Fig. 1D**). A greater reduction was observed for promoter associated with basonuclin (**Fig. 1E**). Within the same cell extract, the amount of UBF-associated rDNA promoter was only slightly reduced. This result suggested that promoter interactions of basonuclin and UBF were differentially regulated.

### Knocking-down basonuclin in HaCaT cells by siRNA

**Figure 2:**
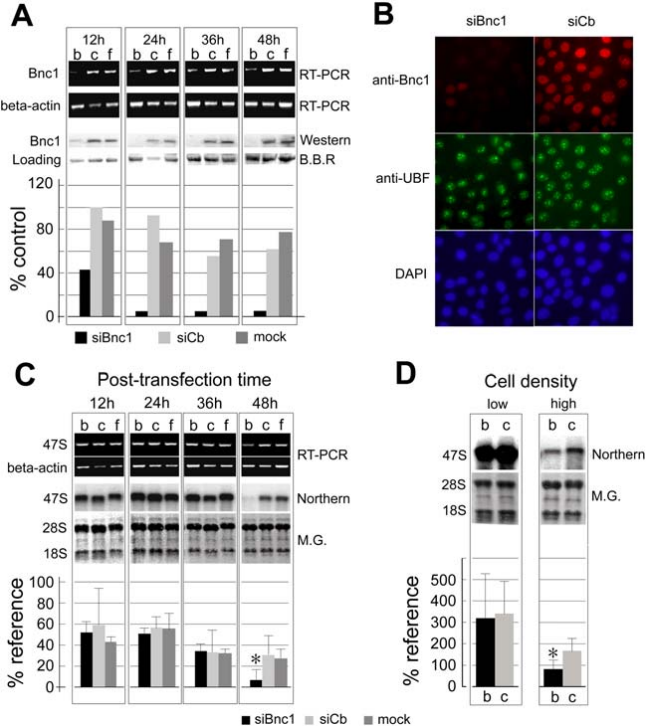
Inhibition of basonuclin expression via siRNA reduces pre-rRNA level in the HaCaT cells. A, HaCaT cells were transfected with siRNA and controls. b, siBnc1 targeting siRNA, c, control siRNA, f, mock transfection (Lipofectamin only). Basonuclin mRNA and protein levels were monitored by PCR and Western blot at four post-transfection time points, 12, 24, 36 and 48 hours. No difference among the experimental groups was detected at 0 h (not shown). A beta-actin RT-PCR served as a control for the quantity and quality (integrity) of RNA. A protein band detected by Brilliant Blue R (B.B.R) was chosen as a loading control because of its consistent quantity in cells receiving different treatments. The protein levels shown in the Western blot were quantified in relation to the loading control (histogram). B, Immunocytochemical staining of transfected cells. HaCaT cells were cultured in chamber slides and transfected with siBnc1-s and siCb-s. Cells were fixed at 48 h post-transfection and stained with anti-basonuclin (anti-Bnc1) and anti-UBF antibodies simultaneously. Microscopic photographs in each column represent the same field, visualized by different secondary antibodies, basonuclin, red (cy5), UBF (FITC) and DNA (DAPI). C, Effect of basonuclin siRNA on the level of 47S pre-rRNA. 47S pre-rRNA was measured by RT-PCR or Northern analysis, with beta-actin and mature rRNA as references, respectively. HaCaT cells were seeded at 1.5×10^5^ cell/35 mm dish and transfected after cells were attached. RNAs were prepared at post-transfection 12, 24, 36 and 48 hours. The treatment groups b, c, and f are as described in (A). The results of Northern analysis was quantified in relation to methylene-green-stained mature rRNA (histogram), n = 3. Note that a reduction of 47S pre-rRNA level was seen only at 48 h post-transfection. M.G., methylene green. D, The effect of basonuclin knock-down could be seen only at high cell density. 47S pre-rRNA level was monitored by Northern analysis. HaCaT cells were seeded at densities: low, 2×10^4^, medium, 5×10^4^ (performed once hence not shown) and high, 1.5×10^5^ and RNA harvested at 48 h post-transfection. The relative level of 47S pre-rRNA to mature rRNA was quantified (histogram) (n = 2). Mock transfection was not performed in every experiment, hence its omission from the quantitative analysis.

To investigate if basonuclin's interaction with rDNA promoter was functional, we knocked-down basonuclin mRNA in HaCaT cells using small interfering RNA (siRNA). We tested two types of siRNA reagents, regular and stealth (Invitrogen), designated respectively as siBnc1-r and siBnc1-s. Each targeting siRNA was matched with a negative control siRNA (siCb-r, and siCb-s), which had the same nucleotide composition of the targeting siRNA but a scrambled sequence. The efficiency of the targeting siRNAs was evaluated by transfecting them into HaCaT cells and monitoring their effect on basonuclin mRNA as compared with the negative controls. The stealth siRNA had lower cytotoxicity and was the more effective, hence was used for all experiments described here. Transfecting siBnc1-s into HaCaT cells decreased basonuclin mRNA level, which reached a minimum at 12 h post transfection but recovered somewhat at 24 h (**Fig. 2A**, RT-PCR) and maintained at a low level during the rest of the experiment. By Western blot, we observed an apparent decrease in basonuclin protein at 12 h post transfection, which fell to less than 5% of the control at 24 h and remained at this level during the observation period (up to 48 h post transfection) (**Fig. 2A**, Western). The reduction in basonuclin was also observed by immunocytochemical staining of transfected cells (**Fig. 2B**). In the majority of HaCaT cells (>90%), nuclear basonuclin was barely detectable 24 h post transfection of siBnc1-s (**Fig. 2B**, siBnc1 column). The protein level of UBF remained unchanged (**Fig. 2B**). The control siRNA (siCb-s) did not affect basonuclin level. Thus, we created a basonuclin knocked-down model in the HaCaT cells.

### Knocking-down basonuclin reduced the level of 47S pre-rRNA at a higher cell density

To investigate basonuclin's role in rRNA synthesis, we monitored the level of precursor rRNA (47S) during the time course of siRNA transfection. HaCaT cells were divided into three groups and transfected with siBnc1-s, siCb-s (control) and mock (i.e., Lipofectamine only) at a cell density of 1.5×10^5^/35 mm dish (the density unit, cell per 35 mm dish, will not be shown hereafter). RT-PCR analysis of total RNA showed that compared with siCb- and mock-transfected cells, the amount of 47S pre-rRNA did not change in siBnc1-s transfected cells at 12, 24 and 36 hours post-transfection (**Fig. 2C**), when basonuclin protein level was barely detectable (**Fig. 2A**). Only at 48 hours post-transfection, a significant decrease in pre-rRNA level in siBnc1-s transfected cells was observed. This result was confirmed by Northern analysis, which showed a 3-5-fold decrease of 47S pre-rRNA at 48 h post-transfection (**Fig. 2C**). The Northern analysis also revealed a decrease of 47S pre-rRNA in the control groups (i.e., siCb-s and mock transfection) at the 48 h, which was not obvious in the RT-PCR assay. This decrease suggested a general decline in pre-rRNA synthesis at the end of the observation period, which was likely due to the higher cell density and a slow-down of cell proliferation (at approximately 4.5×10^5^). To verify this notion, the effect of siBnc1-s was tested at three seeding cell densities, i.e., low, 2×10^4^; medium, 5×10^4^; and high, 1.5×10^5^. These seeding densities produced cell densities mimicking that at 12, 36 and 48 hours post transfection (RNA harvest) when 1.5×10^5^ seeding density was used (**Fig. 2D**). Indeed, Northern analysis confirmed that under normal culture conditions, 47S pre-rRNA synthesis was down-regulated (4–5 fold) when HaCaT culture reached a higher density (**Fig. 2D**). This density-dependent rRNA regulation was consistent with previous observations in 3T6 cells [14]. Furthermore, it verified that only at a higher cell density, the rRNA synthesis reduction in basonuclin-deficient cells could be observed. These results suggested that quantitatively, the rRNA synthesis controlled by basonuclin was minor compared with the massive synthesis required for cell proliferation. Because basonuclin's role in rRNA synthesis could only be observed at a higher cell density, all further analyses (e.g., IP or ChIP) were carried out at a high cell density of 4∼5×10^5^.

### Basonuclin co-precipitated with a Pol I subunit

**Figure 3:**
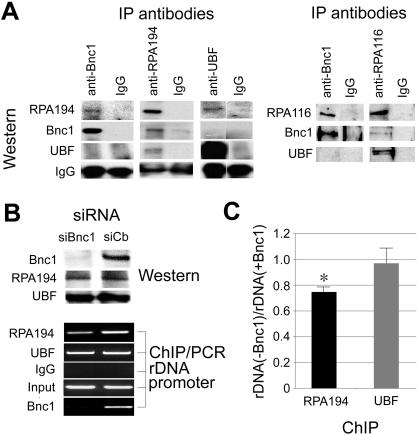
Basonuclin is associated with RPA194 and RPA116, subunits of Pol I. A, Immunoprecipitations detected a basonuclin-RPA194/RPA116 complex. Immunoprecipitations (IP) were performed with extract of HaCaT cells cultured to a high density and analyzed by Western blots. The IP antibodies are indicated above the gel image and that used for Western blots on the left. B, Basonuclin-deficiency reduces the association of RPA194 and rDNA promoter. Basonuclin was knocked-down in HaCaT cells by siRNA and the cells were analyzed by Western for protein levels and by ChIP for association of various factors to rDNA promoter. The Western (upper panel) showed that basonuclin level was reduced to below detection by treatment of siBnc1-s, but the level of RPA194 and UBF was not affected. The lower panel shows the results of PCR analysis of the amount of rDNA promoter precipitated by ChIP. The siRNA reagents the same as indicated in the upper panel. The Western and ChIP antibodies are indicated on the left. C, A quantification of rDNA promoter precipitated by anti-RPA194 in the presence and absence of basonuclin. Shown are qPCR results normalized to the rDNA quantity in the presence of basonuclin. An asterisk indicates p<0.05 (n = 3).

To understand basonuclin's role in rRNA synthesis regulation, we investigated its association with subunits of Pol I (RPA194 and RPA116). Basonuclin was likely an rRNA transcription regulator, but its relation to Pol I had not been ascertained previously. Cell extract was prepared from HaCaT cells cultured to a density of 4.5×10^5^ and analyzed by immunoprecipitation with antibodies against basonuclin, UBF, RPA194 and RPA116 (**Fig. 3A**). Anti-basonuclin antibodies precipitated a complex, which contained RPA194 and RPA116, but not UBF (**Fig. 3A**). The amount of RPA194 and RPA116 precipitated by anti-basonuclin antibody was less than a similar precipitation with anti-RPA194 and anti-RPA116 antibodies (**Fig. 3A**), suggesting that only a fraction of precipitable Pol I subunits was associated with basonuclin. This notion was also supported by the reciprocal precipitation, in which, anti-RPA194 and anti-RPA116 antibodies precipitated complexes containing basonuclin (**Fig. 3A**). The amounts of basonuclin co-precipitated with RPA194 and RPA116 were also low compared with that precipitated directly by the anti-basonuclin antibody (**Fig. 3A**, left panel), suggesting that not all basonuclin was associated with these Pol I subunits. These observations were consistent with the notion that basonuclin also had a role in regulating Pol II-mediated transcription [10]. Interestingly, UBF did not precipitate with basonuclin, or basonuclin with UBF (**Fig. 3A**), suggesting that under the extraction conditions, the two proteins did not form a stable complex. On the other hand, under this condition, UBF was co-precipitated with RPA194 and RPA116, suggesting a more stable interaction. This study provided the first evidence of interaction between basonuclin and Pol I. It also suggested that a sub-population of basonuclin were associated with a sub-population of Pol I and UBF did not stably interact with this basonuclin-Pol I complex.

To investigate if basonuclin-RPA194 complex interacted with rDNA promoter, we examined, via ChIP, whether knocking down basonuclin would affect RPA194's association with the rDNA promoters. HaCaT cells were transfected with siBnc1-s and treated with formaldehyde at a cell density of 4.5×10^5^ (48 h post transfection). The siRNA treatment reduced basonuclin protein level to below detection but did not affect the levels of RPA194 or UBF (**Fig. 3B**, Western). ChIP-PCR analysis showed that knocking-down basonuclin reduced the association of RPA194 to rDNA promoter, but not that of UBF. In the absence of basonuclin, rDNA promoter associated with RPA194 was reduced by approximately 25%, which agreed with our observation in basonuclin-deficiency oocytes [6]. This result supported the notion that basonuclin-RPA194 complex was associated with rDNA promoter and was consistent with the reduced level of 47S pre-rRNA in basonuclin-knock-down HaCaT cells at this cell density (**Fig. 2C, D**).

### Basonuclin-associated rDNA promoter was hypomethylated

**Figure 4:**
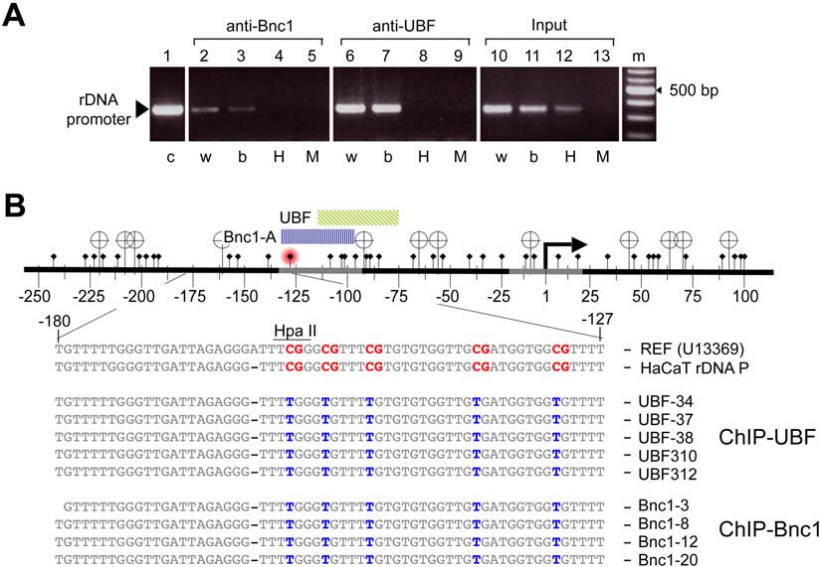
Basonuclin-associated rDNA promoter is hypomethylated. The DNA methylation status of basonuclin-associated rDNA promoter was analyzed by the HpaII/MspI sensitivity assay (A) and by bisulfite sequencing (B). A, A PCR analysis of the integrity of the rDNA promoter after HpaII or MspI digestion. The source of PCR templates is indicated above the gel image, and the treatment received by the templates, below. c, control template (genomic DNA), w, templates were incubated in water, b, in restriction buffer, H, with HpaII, M, with MspI. B, Depicted on top is the region of rDNA amplified by the PCR shown in A. Transcription start site is indicated as a bent arrow and the cis-elements are depicted as gray segments. HpaII/MspI sites are shown as banners and CpG sites, diamond-headed pins. Cytosine at position −132 is marked with a red hallo. The regions of basonuclin and UBF DNase I footprints are indicated by color-coded bars above the DNA. Listed below are DNA sequences from the region in between −180 to −126. The reference (U13369) and HaCaT rDNA promoter consensus sequences (the first two lines, respectively) are shown as they are sequenced after bisulfite treatment; i.e., all CpGs (in red) are assumed to be methylated and the rest of the Cs are converted to Ts due to the bisulfite treatment. The rest nine sequences are actual bisulfite sequencing data from five UBF-associated promoters and four basonuclin-associated promoters. The presence of a T (in blue) in the CpG position signifies that the CpG was not methylated.

The transcription activity of rDNA had been shown to correlate with DNA methylation within the promoter (for a recent review, [15]). We investigated, by an Hpa II sensitivity assay and by bisulfite sequencing, if basonuclin- and UBF-associated rDNA promoters were differentially methylated. ChIP DNAs were digested with either Hpa II (methylation sensitive) or its isoschizomer, Msp I (CpG methylation insensitive), and the digested DNAs were used as templates for PCR amplification of the promoter region. Hpa II sensitivity assay showed that both basonuclin- and UBF-associated promoters were hypomethylated (**Fig. 4A**). The same assay also showed that approximately one half of the genomic rDNA promoters was hypermethylated, as reported previously [16]. In mouse, the critical methylation site, which abolished UBF binding to the promoter, was identified to be the cytosine at −133 [17]. In human, there was also a CpG at −132, which overlapped with the high-affinity binding site of basonuclin (**Fig. 4B**). Bisulfite sequencing confirmed the overall hypomethylation status of both basonuclin- and UBF-associated promoters (**Fig. 4B**). It also showed that the CpG at −132 was not methylated. These results supported the notion that basonuclin was associated with actively transcribed rDNA.

### HaCaT rDNA promoters contain single nucleotide polymorphisms with promoter-specific distribution

**Figure 5:**
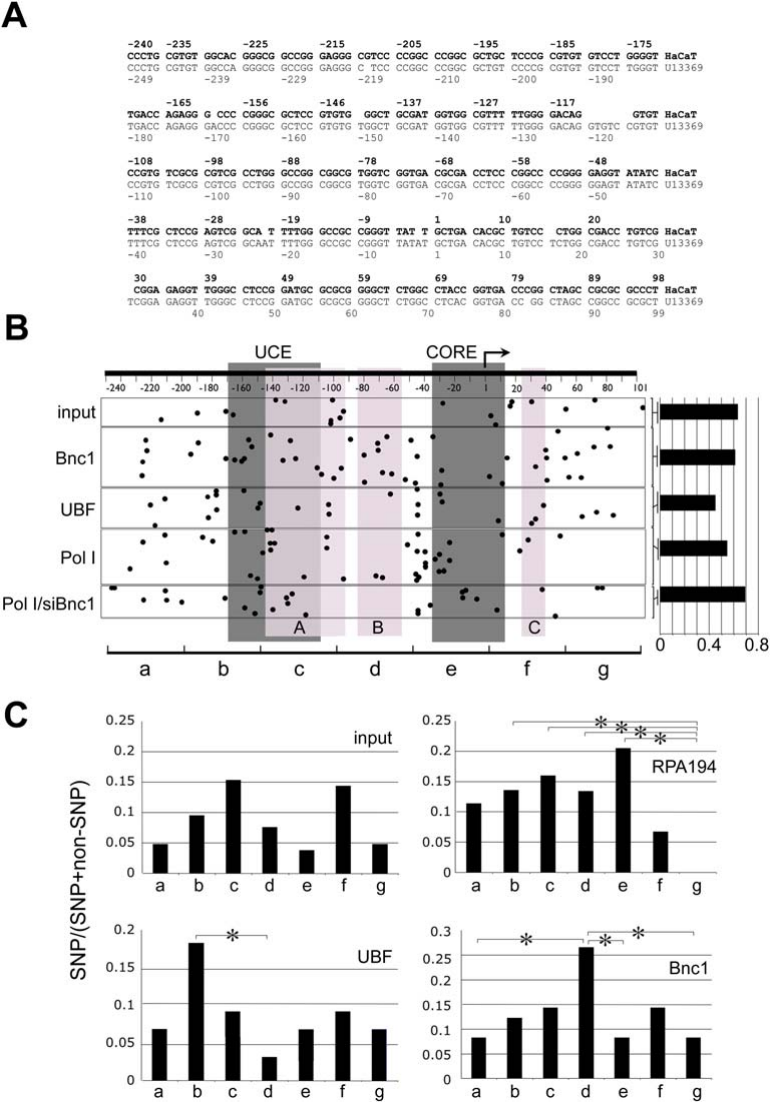
Single nucleotide polymorphism (SNP) in HaCaT rDNA promoters. A, A consensus DNA sequence of HaCaT cell rDNA promoters. The consensus (in bold letters) was deduced from the sequence of 181 HaCaT rDNA promoter clones, which were obtained from PCR-amplified rDNA promoters. Twenty-one of the clones were obtained directly from genomic DNA, the rest were isolated from ChIP DNA precipitated by anti-basonuclin (49 clones), anti-UBF (44 clones), anti-RFA194 in the presence (44 clones) and absence of basonuclin (23 clones). A human rDNA promoter sequence (U13369) is listed below the HaCaT consensus. The two sequences are aligned according to their transcription start site (+1). B, A tabulation of SNPs in various ChIP groups. A generic rDNA promoter is depicted at the top with transcription start site (bent arrow), and the cis elements (dark gray boxes) indicated. UCE, up-stream control element and CORE, the core element. Nucleotide coordinates are shown below the DNA. The three basonuclin binding sites (light gray boxes labeled A, B, C) are indicated. SNPs are depicted as dots. The horizontal position of each dot indicates its location in the rDNA promoter, and its vertical position, the ChIP groups as indicated on the left. Input, a collection of randomly picked genomic rDNA promoters, Bnc1-, UBF- and RPA194- (Pol I) associated promoters; Pol I/siBnc1, RPA194-associated promoters in basonuclin-deficient cells. Not all clones contained SNP and the percentage of SNP-containing clones in each ChIP group is shown by a histogram on the right. For a statistical analysis of the SNP frequency, see Table 1. C, For analyzing the regional SNP variations, the promoter is divided into seven 50-base non-overlapping regions, which were named alphabetically, as indicated at the bottom of (B), a, −250 to −201, b, 200 to 149, etc. The regional SNP frequencies are plotted for the input and three ChIP groups, RPA194, UBF and Bnc1. Statistically significant differences are indicated (*, *p*<0.05).

The hypothesis that basonuclin and UBF resided in different RPA194/RPA116 complexes predicted that they were not associated with the same rDNA promoter at the same time. To test this hypothesis and to characterize basonuclin- and UBF-associated rDNA promoters, basonuclin-, UBF-, and RPA194-associated chromatins were purified in two-rounds of immunoprecipitation from HaCaT cells. rDNA promoter (−248 to +100) was PCR-amplified from the precipitated chromatin and cloned. Randomly picked clones were sequenced. Sequence analysis showed several consistent variations between the HaCaT rDNA promoter and the human rDNA promoter sequence in the GenBank (Accession No. U13369). For our analysis, these variations were uninformative because of their ubiquitous presence in all cloned HaCaT rDNA promoters. Therefore, a consensus sequence of HaCaT rDNA promoter was derived from 181 individual promoter sequences (**Fig. 5A**). This consensus sequence served as a baseline for identifying single nucleotide polymorphisms (SNP) in rDNA promoters associated with basonuclin, UBF and RPA194. Of the 181 clones sequenced, 106 contained SNPs. An additional 10 clones contained only small deletions and insertions, which were all in the transcribed sequence and hence not included for the SNP analysis. Overall, there were 167 SNPs in the 106 clones, but no SNP was consistently associated with promoters precipitated by a particular antibody (ChIP group) (**Fig. 5B**). The SNP frequencies in promoters precipitated by various antibodies differed (**Fig. 5B**, histogram); however, the differences were not statistically significant (**Table 1**). Next, we divided the promoter into seven 50-base regions and examined the regional SNP distribution within each ChIP group (**Fig. 5B, C**). The SNP frequency of each region was scored and cross-compared with other regions within the same promoter. These regional SNP frequencies showed characteristic differences in promoters associated with RPA194, basonuclin and UBF, but not in the control (input)(**Fig. 5C**). For example, in basonuclin-associated promoters, ∼20.0% of SNPs (10/49) were found in between nucleotides −100 to −51 (**Fig. 5C**, region d), which was significantly higher than the other regions within the same promoter. More interestingly, in the same region of rDNA promoters isolated from genomic DNA (input), no SNP (0/21) was present. Similarly, in UBF-associated promoters, the same region contained only one SNP (1/44 or 2.3%), and this value was significantly different from that of the basonuclin-associated promoter (*p*<0.05) (**Fig. 5C**). This analysis strongly suggested that regional SNP frequency was transcription-factor-specific.

**Table 1:** *P* values of pair-wise comparison of SNP frequency in rDNA promoters obtained from ChIP assays.

	Input	Bnc1	UBF	RPA194	RPA194 w/o Bnc1
Input		0.957	0.214	0.828	0.592
Bnc1			0.128	0.834	0.492
UBF				0.200	0.060
RPA194					0.400

Nomenclatures are as described in Fig. 5.

### Basonuclin and UBF were associated with different subsets of rDNA promoters

**Figure 6:**
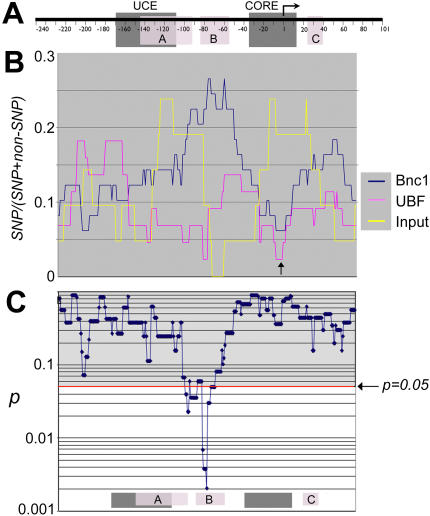
A comparison of the local SNP frequencies in rDNA promoters. A, Depicted is a generic rDNA promoter, to which graphs in B and C are aligned. B, The frequency of SNP was scored by scanning the promoter sequence with a 50-base window. The scores are expressed as the ratio of SNP/(SNP+non-SNP). Each SNP score is aligned to A, using the center nucleotide of each window. Shown are SNP scores of three promoter sets: Bnc1, UBF and the input. An arrow indicates the low SNP frequency near the transcription start site in basonuclin- and UBF-associated promoters, but not in the input. In C, a series of pair-wise chi-square tests were performed on the corresponding SNP frequencies of basonuclin- and UBF-associated promoters. The resulting *p* values are plotted in alignment with A and B. As a reference, the positions of cis-elements as well as basonuclin binding sites are shown at the bottom of panel B.

To explore further the characteristic SNP distributions among the ChIP groups, we scanned the promoter region to score SNPs using several “window” sizes (i.e., 40, 50, 60, 100 bases). The scanning approach avoided the potential bias in dividing the promoters in non-overlapping regions. The window size smaller than 40 was not used because it might not contain sufficient number of SNPs for statistical analysis. To scan, the 50-base window was shifted in one nucleotide increment. The SNP scores were expressed as the ratio of SNP/(SNP+non-SNP) for each window size and plotted in alignment with the promoter sequence, using the center nucleotide to register each window (**Fig. 6A**). A prominent feature of basonuclin and UBF promoters was the low SNP frequency in the windows centered on the transcription start site (TSS, arrow in **Fig. 6B**), which was consistent with the importance of this sequence to rRNA transcription [18]–[20].

**Figure 7:**
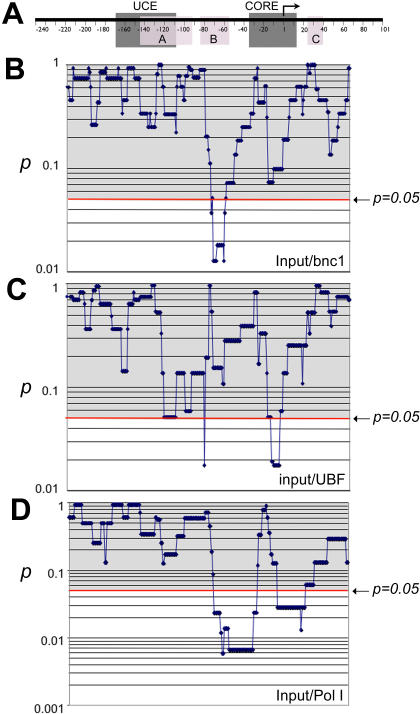
A statistical pair-wise comparison of local SNP frequency between the randomly picked genomic rDNA promoter and the promoters associated with basonuclin, UBF, or RPA194. A, a generic rDNA promoter, to which all the other panels (B, C, D) are aligned. The notations on the rDNA promoter are as described in Fig. 5A. In panel B, C, D, *p* values were calculated by a chi-square test and plotted. The notation and graphic depictions are as described in Fig. 5B. B, input vs. basonuclin, C, input vs. UBF, D, input vs. RPA194.

A chi-square (3×2) test was used to compare the SNP frequencies between the promoters (**Fig. 6C**). The null hypothesis was that the SNP frequency was random and not related to the promoter type. The chi-square tests showed that for all window sizes (i.e., 40, 50, 60 and 100 bases), the results were similar, and the larger the window size, the lower the resolution. We report here the results obtained with a window size of 50 bases. The chi-square tests showed that generally, for approximately two thirds of nucleotide positions (i.e., between windows centered at −245 to −100 in the promoter, as well as between windows centered at +10 to +100 in the transcribed region), the null hypothesis was accepted (**Fig. 6** **,** **7** **,** **8**, grey regions above the red line). However, the tests also revealed that in windows centered at −100 to +10, the null-hypothesis was rejected (*p*<0.05) in various chi-square testings, which meant that regional SNP frequencies were indeed related to transcription factor/promoter interaction (**Fig. 6C** and **7**). As mentioned earlier, basonuclin-associated promoters showed a higher SNP frequency than that associated with UBF in between nucleotides −100 to −51. The chi-square test confirmed this conclusion by showing that the two types of promoters differed in windows centered at −94 to −65, and pinpoint the difference to be centered within basonuclin binding site B (**Fig. 6C**).

**Figure 8:**
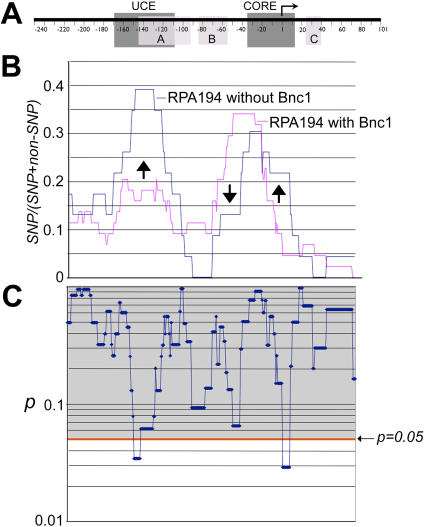
A comparison of RPA194-associated promoters in the presence and absence of basonuclin. A, A generic rDNA promoter is depicted as described in Fig. 5. B, SNP frequencies of RPA194-associated promoters are plotted as described in Fig. 5. Arrows show the region as well as the direction of changes caused by basonuclin-deficiency. C, Pair-wise chi-square tests on SNP frequencies shown in B were performed. The resulting *p* values are plotted in alignment with panels in A and B. The notations are as described in Fig. 4 and 5.

Similarly, compared with randomly picked rDNA promoters (input), basonuclin-associated promoters showed a significantly higher SNP frequency in windows centered at −68 to −56 (**Fig. 7B**, input/Bnc1). On the other hand, compared with the input, the UBF-associated promoters showed a significantly lower SNP frequency in windows centered at −7 to +2, and a higher SNP frequency in windows centered at −77 (**Fig. 7C**). Remarkably, these characteristic SNP distributions of basonuclin- and UBF-associated promoters were reflected in the RPA194-associated rDNA promoters, which showed a significantly lower SNP frequency around the TSS (−10 to +20) and a higher frequency between windows centered at −20 to −70 (**Fig. 7D**, input/Pol I). These analyses strongly suggested that basonuclin and UBF were associated with different subsets of rDNA promoters, and both sets of promoters were associated with RPA194.

We also analyzed the effect of basonuclin-deficiency on promoters associated with RPA194 (**Fig. 8**). Promoters associated with RPA194 showed a characteristic SNP distribution, in which a high SNPs frequency was detected in between −50 to −10 (**Fig. 8B**). Knocking down basonuclin clearly modified this distribution. This modification was consistent with the reduction of RPA194's association with rDNA promoter in basonuclin-deficient HaCaT cells (**Fig. 3B**), for such reduction would change the promoter composition associated with RPA194. Furthermore, the reduction of SNP frequency in the region (−50 to −70) was also consistent with the loss of promoters associated with basonuclin, which had a higher SNP frequency in this region (**Fig. 6B** and **Fig. 8B**, arrow pointing down). This reduction was accompanied by an increase in SNP frequency around −20 to +20 (**Fig. 8A**, arrow pointing up), including TSS, suggesting that this increase was not due to an increase in the proportion of UBF-associated promoter, which had a low SNP frequency −20 to +1 (**Fig. 6A**). In basonuclin-deficient HaCaT cells, the most prominent change in RPA194-associated promoters was an increase in SNPs upstream the promoter, in the region between −170 and −120. This change might be brought about by promoters previously not associated with RPA194.

## Discussion

We present evidence that basonuclin is associated with rDNA promoter in vivo. We demonstrate this point in HaCaT cells by ChIP assays. Similar results were obtained with mouse testicular cells, using an independent anti-mouse basonuclin antibody (Zhang and Tseng unpublished), which will be reported elsewhere. Our mapping experiment suggests that basonuclin is mainly associated with the promoter of rDNA, and to a lesser extend, with the ITS-1, but not with IGS. This distribution is different from that of UBF [13], but similar to that of c-Myc [21]. The interaction of basonuclin and rDNA promoter is likely functional because knocking down basonuclin in HaCaT cells reduces both RPA194's association with rDNA promoter and the amount of 47S pre-rRNA. Similarly, we showed previously that knocking down basonuclin in mouse oocytes reduced the number of Pol I transcription foci [6].

Our immunoprecipitation study provides the first evidence of a protein complex containing basonuclin, RPA194 and RPA116, but not UBF. Furthermore, only a fraction of precipitable RPA194 and RPA116 is pulled down by the anti-basonuclin antibody. This observation is supported by the result that knocking-down basonuclin partially reduces RPA194's association with rDNA promoter but does not affect that of UBF. We were not able to knock-down nucleolar UBF via siRNA (Zhang and Tseng, unpublished), which preclude the experiment examining how absence of UBF would affect basonuclin's association with rDNA promoters. The notion that basonuclin forms complex with a fraction of Pol I is also supported by the magnitude of reduction in pre-rRNA level caused by basonuclin-deficiency. This reduction is smaller compared with proliferation-related rRNA synthesis. These observations suggest that (i) proliferation-related rRNA synthesis, which is regulated via UBF, TIF-1A/Rrn3p, Rb and a number of other factors [14], [22]–[31], is dominant during cell cycle; (ii) the rRNA synthesis that requires basonuclin becomes significant after the down regulation of proliferation-related synthesis when keratinocytes become quiescent.

Our previous observation suggested that basonuclin regulates a subset of rDNA [6]. The results discussed above are also consistent with this notion. Sequence analysis of HaCaT rDNA promoters precipitated by various antibodies provides additional support. Mammalian cells possess several hundreds of rDNA transcription units and little is known about the SNP of their promoters. Our sequencing data show that rDNA promoters of HaCaT cells contain numerous SNPs, which are not likely a result of the standard PCR and cloning artifact, because the same procedure produced very few SNPs in mouse rDNA promoters (Zhang and Tseng, unpublished). More likely, these SNPs are a result of the interplay between random mutation and selection during the long culture history of HaCaT cells. In other words, rDNA array in cultured cells does not undergo repair or homogenization that takes place during meiosis in gametogenesis. The stochastic origin of SNPs in HaCaT rDNA promoter is supported by our observation that the distribution of these SNPs is random in the 5′ transcribed region of pre-rRNA, which does not have a known promoter function, but these distributions show significant characteristics in regions critical for promoter and transcription factor function. The most important finding relevant to our proposal is that the distribution of SNPs suggests that basonuclin-associated rDNA promoters differ from that associated with UBF, as well as from a random sampling of genomic rDNA promoters.

Our study sheds light on several issues in basonuclin research. The first concerns whether basonuclin and UBF bind to the same rDNA promoter molecule simultaneous. Previously, DNase I footprinting showed that in vitro basonuclin interacted with three highly conserved sites (binding site A, B and C) within human and mouse ribosomal RNA gene (rDNA) promoters [3]–[5]. Binding site A has the highest affinity to basonuclin and overlaps partially with the footprint of UBF [3]–[5]. This overlap raises a question if the two regulators interact with the promoter cooperatively or competitively [5]. The data presented here favor the notion that basonuclin and UBF are associated with different rDNA promoter molecules. This conclusion is supported by the characteristic SNP distributions within each rDNA promoter, as well as by the observation that basonuclin and UBF form separate RPA194/RPA116 complexes. Although our promoter sequence comparison cannot completely exclude co-existence of basonuclin and UBF on some promoters, our data strongly suggest that their promoter preferences differ. It should be pointed out that our data do not suggest how basonuclin and UBF recognize their own promoter subsets. We have not detected any consistent sequence features in the promoter region. The lack of consistent sequence features precludes a sequence-based recognition. Possibly, regions outside basonuclin and UBF binding sites, other protein factors, chromatin structure and epigenetic modifications provide the basis for discerning the different promoter subsets [15], [32]. This issue warrants future investigation.

Another issue concerns a lack of protein sequence conservation in basonuclin's middle pair zinc fingers (zinc fingers 3 and 4) [10], [33]. This lack of evolutionary conservation of middle pair is in contrast to the high degree of conservation seen in the N-terminal pair (zinc fingers 1 and 2) and the C-terminal pair (zinc fingers 5 and 6) from fish to human [9]. The interpretation was that zinc fingers 3 and 4 were not required for sequence-specific DNA binding. Our analysis supports the interpretation that the sequence-specificity of the middle pair zinc fingers is relaxed. The overall random distribution of SNPs in HaCaT rDNA promoters makes these SNPs a probe for functional constraints on a DNA sequence (i.e., similar to artificial mutagenesis). If a promoter region accumulates random SNP, it is likely that the sequence in that region is not critical for promoter function. It is therefore unlikely a coincidence that basonuclin binding site B (i.e., the middle binding site) accumulates random SNPs, whereas binding sites A and C have lower SNP frequency (**Fig. 5A**). Similarly, the absence of SNP accumulation in the same region (i.e., −100 to −60) in the UBF-associated promoters suggests that the sequence in that region is important for Pol I complex that contains UBF, though this sequence requirement is unlikely due to UBF, which binds to a different site [34]–[36]. Conceivably, another DNA binding factor in the UBF-Pol I complex interacts with this region [32]. Therefore, the higher SNP frequency in certain regions is likely the results of lack of selective pressure for that region, and is not a requirement for a particular interaction.

The third issue relates to our inability to detect pre-rRNA changes in basonuclin-deficient oocytes, despite the fact that the number of Pol I transcription foci was clearly reduced [6]. In light of our present study, this discrepancy is likely due to the high level of rRNA transcription in the oocytes, which might mask the small drop in the 47S pre-rRNA level due to loss of basonuclin. We encountered that same problem in our HaCaT basonuclin knock-down model. The massive proliferation-related rRNA synthesis masked the effect of loss of basonuclin. It was only when proliferation-related synthesis subsided in a confluent culture, the amount of rRNA synthesis controlled by basonuclin became significant. Our study shows an inadequacy in the current method of assaying rRNA synthesis, which cannot readily detect changes in the expression of a subset of rDNA.

Although it has been well recognized that different cell types have distinctive requirement for rRNA synthesis, cell-type specific regulation has not been well studied [37]. The first cell-type- and developmental-stage-specific regulatory mechanism for rRNA synthesis was discovered from studying Xenopus oogenesis [38], [39]. Cell lineage-specific regulation of rRNA synthesis was noted in a recent report on Runx2 [40]. Runx2 is a member of the Runt-related transcription factor family, which establishes and maintains cell identity. Runx2 was shown to repress rRNA transcription by affecting Pol I complex and local chromatin modification at the rDNA promoter region. It was proposed that lineage-specific control of ribosomal biogenesis is a fundamental function of transcription factors that govern cell fate [40]. Another study of mouse TIF-1A (a homolog of yeast Rrn3p) also suggested the existence of yet recognized regulatory pathways for rRNA synthesis. TIF-1A, a basal Pol I transcription factor, is essential for rRNA transcription in cultured cells and a key mediator for proliferation-related regulation of Pol I activity [31], [41]–[43]. It is therefore surprising that TIF-1A -/- embryo could survive to E9.5 and developed all three germ layers as well as extra-embryonic tissues [44]. This observation raised an interesting question regarding rRNA transcription requirement in early embryogeneisis and the possibility of alternative rRNA synthesis pathways that differ from the one currently understood. Our results support the notion that other rRNA synthesis regulatory pathways exist and suggest that basonuclin is a component of one of these pathways.

Because rDNA is a multicopy gene entity, its transcriptional output can be regulated by transcription rate of individual copies, or the number of copies transcribed. Regulation of the size of rDNA pool that is actively transcribed has not been well studied [45]. A particularly interesting feature of cell-type-specific regulation of rRNA synthesis may relate to the number of rDNA transcribed [37]. A series of previous cytogenetic studies revealed that individual rDNA clusters could be regulated independently in different cell types and in response to serum stimulation [46]–[48]. These studies concluded that transcription activity is regulated at the level of single rDNA cluster and transcription rate can be modulated by activating or repressing individual clusters according to the metabolic requirements of the cell. In other words, cell-type-specific regulation of rRNA synthesis involves differential usage of a subset of rDNA. This notion was re-enforced by a latter study examining the number of Pol I transcription foci in different species and cell types [49]. Our data corroborate these early results that cell-type-specific regulation of rRNA synthesis relates to differential transcription from subsets of rDNA. Future studies should focus on understanding the substructure in rDNA array and on molecular isolation of rDNA transcription units regulated by cell-type-specific factors.

## Materials and Methods

### Cell culture

HaCat cells were grown on 100 mm-diameter culture dishes in DMEM supplemented with 10% bovine calf serum at 37°C and 5% CO_2_. For immunocytochemistry, HaCaT cells were grown on chamber slides (Nunc Inc., Naperville, IL)

### Immunoprecipitation

The anti-human-Bnc1 antibody was described in [3]. The anti-RFA116 antibody was a generous gift from Ingrid Grummt [24]. Commercial antibodies were obtained from Santa Cruz Biotechnology Inc. (anti-UBF H-300, sc-9131; anti-RPA194 H-300, sc-28714; anti-Wilm's Tumor antigen or WT1 F-6, sc-7385). Cells were lysed in the RIPA buffer, i.e., 1% NP-40, 0.5% sodium deoxycholate, 0.1% SDS, 5 mM EDTA, 0.5 mM phenylmethanesulfonyl fluoride or PMSF, 100 ng/ml protease inhibitor cocktail from Roche Applied Sciences (Penzberg, Germany) in 1× PBS. Immunoprecipitation was performed as previously described [10]. Briefly, precleared cell lysates were incubated with 2 µg of antibodies at 4°C overnight, then precipitated with Protein A Sepharose beads followed by washing with a low sodium washing buffer, a high sodium washing buffer and 1× PBS. Immunocomplexes were separated by electrophoresis on a SDS-polyacrylamide gel (SDS-PAGE).

### Knocking down basonuclin by small interfering RNA

Small interfering RNAs (siRNA) and the corresponding negative controls were designed by a web-based software BLOCK-iT RNAi Designer (Invitorgen, Carlsbad, CA). The siRNAs are:

Stealth-siRNA (si-Bnc-s) targets human basonuclin sequence at 1220 to 1224: 5′-GCCGTCCACTTGAAGATCAAGCATA-3′, Negative control of stealth-siRNA (si-Cb-s):5′-GCCACCUUCAAGUAGAACCGGUAUA-3′. All siRNA reagents were obtained from Invitrogen.

For transfection, cells were seeded at 1.5×10^5^/well in a 6-well plate one day before transfection, and transfected with annealed siRNA using Lipofectamine 2000 (Invitrogen) in a procedure recommended by the manufacturer. Cells were harvested at the indicated post-transfection time for RNA and protein analysis.

### Western blotting

Protein samples were electrophoresed on SDS-PAGE and electro-transferred onto polyvinylidene difluoride (PVDF) membranes (Immobilon-P, Millipore, Bedford, MA). Each PVDF blot was incubated with a primary antibody (see Immunoprecipitation for source) at 4°C overnight. The primary antibodies were visualized by horseradish peroxidase-conjugated secondary antibodies (1:10000 dilution) and detected with an ECL Western blotting system (Amersham Biosciences, Piscataway, NJ). Prestained molecular weight standards (New England Biolabs, MA) were used in estimating the apparent molecular weight.

### Northern analysis of pre-rRNA

Total RNA was isolated from cultured HaCaT cells using Trizol reagent (Invitorgen, Carlsbad, CA) at indicated post-transfection time. Equal amount of RNA from each time point was electrophoresed on agarose gel and transferred to a Hybond-N+membrane (Amersham Biosciences, Piscataway, NJ). The blot was probed with ^32^P-labeled 5′-ETS sequence (the first 300 bp of 47S pre-rRNA).

### Immunocytochemistry

Cells were cultured in 2-well chamber slides and fixed by methanol and acetone as described in [3]. Anti-Bnc1 and anti-UBF antibodies were diluted in 3% BSA/PBS and incubated with cells overnight at 4°C. The primary antibodies were visualized by fluorescently labeled secondary antibodies, i.e., a cy5-labeled goat-anti-rabbit for Bnc1 or a FITC-labeled goat-anti-mouse for UBF (sc-3844 and sc-3699, Santa Cruz Biotechnology Inc.). The antibodies were diluted as instructed by the manufacturer.

### Chromatin Immunoprecipitation (ChIP) assay

ChIP was performed as previously described [10]. Briefly, HaCaT cells were fixed with 1% formaldehyde for 10 minutes at 37°C. The fixed cells were lysed in the RIPA buffer and sonicated on ice with an ultrasonic sonicator (Dr. Hielscher UP 100H) at amplitude = 1 and duty cycle = 100% in 12 one-minute pulses. The sonicated cell lysates were precleared with Protein A Sepharose beads (Amersham Biosciences, Piscataway, NJ) pre-treated with salmon sperm DNA and BSA. Chromatin was precipitated by 2 µg of antibodies as indicated. The immuno-complexes were absorbed with 75 µl of salmon-sperm-DNA- and BSA-treated Protein A Sepharose beads, and washed for 5 times with each immunoprecipitation buffer as previously described [10]. The ChIP was performed once more and immuno complexes were eluted with 300 µl of elution buffer (1% SDS, 0.1 M NaHCO_3_). Cross-link was reversed. DNA was extracted by phenol/chloroform.

### ChIP-Southern

DNA eluted from ChIP was linked with a primer (5′-TCGACCCACGCGTCCG), which served as the priming sites for PCR. PCR products were separated by electrophoresis on an agarose gel and transferred to a Hybond-N+membrane. The blot was probed with ^32^P-labeled rDNA promoter (−241 to 160).

### ChIP-PCR, Cloning and Sequencing

The rDNA promoter (from −248 to +100 of human rDNA U13369) was amplified by PCR using the DNA eluted from ChIP as a template. The primers used in examining the presence of ITS 1 and IGS were those designated H8 and H23/27 in [13]. Standard PCR reactions were performed for 25 cycles following a 5-minute incubation at 72°C. Each cycle consisted of 30 seconds at 94°C, 30 seconds at 66°C, and 30 seconds at 72°C. The PCR products were cloned into a plasmid (pGEM-T Easy Vector, Promega, Madison, WI) and sequenced by the DNA Sequencing Facility in the Department of Genetics at PENN. Bisulfite sequencing was done with an EZ DNA methylation Kit (ZYMO Research Co., Orange, CA) following manufacturer's instructions. Sequence alignment was first performed by a web-based program (http://bioinfo.genopole-toulouse.prd.fr/multalin/) [50] and the machine alignment was then refined manually.

### Statistical analysis of SNPs

Three types of analyses were performed. **1**) Tests of dependency of SNP frequencies among ChIP groups in the entire promoter region (**Table 1**); **2**) tests of dependency of SNPs among non-overlapping sub-regions of each ChIP group (**Fig. 4C**); and **3**) tests of dependency of SNP frequencies among ChIP groups by sampling with a scanning window. For the chi-squared test, the *p*-value of independence was computed from 2×2-contingency tables (one degree of freedom for **Fig. 5**, **7** and **8**) or from 3×2-contigency tables (2-degree of freedom for **Fig. 6**). For the large sets of *p*-values (295 *p*-values, **Fig. 5** **,** **6** and **7**), all values were examined by a *q*-value program [51] (http://faculty.washington.edu/jstorey/qvalue/index.html), which calculates a multiple-test correction. All significant *p*-values passed the *q*-value threshold (False Discovery Rate, FDR) of 0.20.

## References

[1] Tseng H (1998). Basonuclin, a zinc finger protein associated with epithelial expansion and proliferation.. Front Biosci.

[2] Green H, Tseng H, Iuchi S, Kuldell N (2005). Basonuclin: A zinc finger protein of epithelial cells and reproductive germ cells.. Zinc Finger Proteins: From Atomic Contact to Cellular Function.

[3] Tseng H, Biegel JA, Brown RS (1999). Basonuclin is associated with the ribosomal RNA genes on human keratinocyte mitotic chromosomes.. J Cell Sci 112 Pt.

[4] Iuchi S, Green H (1999). Basonuclin, a zinc finger protein of keratinocytes and reproductive germ cells, binds to the rRNA gene promoter.. Proc Natl Acad Sci U S A.

[5] Tian Q, Kopf GS, Brown RS, Tseng H (2001). Function of basonuclin in increasing transcription of the ribosomal RNA genes during mouse oogenesis.. Development.

[6] Ma J, Zeng F, Schultz RM, Tseng H (2006). Basonuclin: a novel mammalian maternal effect gene.. Development.

[7] Boukamp P, Petrussevska RT, Breitkreutz D, Hornung J, Markham A (1988). Normal keratinization in a spontaneously immortalized aneuploid human keratinocyte cell line.. J Cell Biol.

[8] Tseng H, Green H (1994). Association of basonuclin with ability of keratinocyte to multiply and with absence of terminal differentiation.. J Cell Biol.

[9] Iuchi S, Green H (1997). Nuclear localization of basonuclin in human keratinocytes and the role of phosphorylation.. Proc Natl Acad Sci U S A.

[10] Wang J, Zhang S, Schultz RM, Tseng H (2006). Search for basonuclin target genes.. Biochem Biophys Res Commun.

[11] Morris JF, Madden SL, Tournay OE, Cook DM, Sukhatme VP (1991). Characterization of the zinc finger protein encoded by the WT1 Wilms' tumor locus.. Oncogene.

[12] Rauscher FJ (1993). The WT1 Wilms tumor gene product: a developmentally regulated transcription factor in the kidney that functions as a tumor suppressor.. Faseb J.

[13] O'Sullivan AC, Sullivan GJ, McStay B (2002). UBF binding in vivo is not restricted to regulatory sequences within the vertebrate ribosomal DNA repeat.. Mol Cell Biol.

[14] Hannan KM, Kennedy BK, Cavanaugh AH, Hannan RD, Hirschler-Laszkiewicz I (2000). RNA polymerase I transcription in confluent cells: Rb downregulates rDNA transcription during confluence-induced cell cycle arrest.. Oncogene.

[15] Grummt I, Pikaard CS (2003). Epigenetic silencing of RNA polymerase I transcription.. Nat Rev Mol Cell Biol.

[16] Conconi A, Widmer RM, Koller T, Sogo JM (1989). Two different chromatin structures coexist in ribosomal RNA genes throughout the cell cycle.. Cell.

[17] Santoro R, Grummt I (2001). Molecular mechanisms mediating methylation-dependent silencing of ribosomal gene transcription.. Mol Cell.

[18] Learned RM, Tjian R (1982). In vitro transcription of human ribosomal RNA genes by RNA polymerase I.. J Mol Appl Genet.

[19] Jones MH, Learned RM, Tjian R (1988). Analysis of clustered point mutations in the human ribosomal RNA gene promoter by transient expression in vivo.. Proc Natl Acad Sci U S A.

[20] Haltiner MM, Smale ST, Tjian R (1986). Two distinct promoter elements in the human rRNA gene identified by linker scanning mutagenesis.. Mol Cell Biol.

[21] Grandori C, Gomez-Roman N, Felton-Edkins ZA, Ngouenet C, Galloway DA (2005). c-Myc binds to human ribosomal DNA and stimulates transcription of rRNA genes by RNA polymerase I.. Nat Cell Biol.

[22] Cavanaugh AH, Hempel WM, Taylor LJ, Rogalsky V, Todorov G (1995). Activity of RNA polymerase I transcription factor UBF blocked by Rb gene product [see comments].. Nature.

[23] Voit R, Kuhn A, Sander EE, Grummt I (1995). Activation of mammalian ribosomal gene transcription requires phosphorylation of the nucleolar transcription factor UBF.. Nucleic Acids Res.

[24] Voit R, Schafer K, Grummt I (1997). Mechanism of repression of RNA polymerase I transcription by the retinoblastoma protein.. Mol Cell Biol.

[25] Voit R, Hoffmann M, Grummt I (1999). Phosphorylation by G1-specific cdk-cyclin complexes activates the nucleolar transcription factor UBF.. Embo J.

[26] Voit R, Grummt I (2001). Phosphorylation of UBF at serine 388 is required for interaction with RNA polymerase I and activation of rDNA transcription.. Proc Natl Acad Sci U S A.

[27] Hannan KM, Hannan RD, Smith SD, Jefferson LS, Lun M (2000). Rb and p130 regulate RNA polymerase I transcription: Rb disrupts the interaction between UBF and SL-1.. Oncogene.

[28] Ciarmatori S, Scott PH, Sutcliffe JE, McLees A, Alzuherri HM (2001). Overlapping functions of the pRb family in the regulation of rRNA synthesis.. Mol Cell Biol.

[29] Stefanovsky VY, Pelletier G, Hannan R, Gagnon-Kugler T, Rothblum LI (2001). An immediate response of ribosomal transcription to growth factor stimulation in mammals is mediated by ERK phosphorylation of UBF.. Mol Cell.

[30] Lin CY, Tuan J, Scalia P, Bui T, Comai L (2002). The cell cycle regulatory factor TAF1 stimulates ribosomal DNA transcription by binding to the activator UBF.. Curr Biol.

[31] Mayer C, Bierhoff H, Grummt I (2005). The nucleolus as a stress sensor: JNK2 inactivates the transcription factor TIF-IA and down-regulates rRNA synthesis.. Genes Dev.

[32] Beckmann H, Chen JL, O'Brien T, Tjian R (1995). Coactivator and promoter-selective properties of RNA polymerase I TAFs.. Science.

[33] Matsuzaki K, Iuchi S, Green H (1997). Conservation of human and mouse basonuclins as a guide to important features of the protein.. Gene.

[34] Bell SP, Jantzen HM, Tjian R (1990). Assembly of alternative multiprotein complexes directs rRNA promoter selectivity.. Genes Dev.

[35] Pikaard CS, Smith SD, Reeder RH, Rothblum L (1990). rUBF, an RNA polymerase I transcription factor from rats, produces DNase I footprints identical to those produced by xUBF, its homolog from frogs.. Mol Cell Biol.

[36] Copenhaver GP, Putnam CD, Denton ML, Pikaard CS (1994). The RNA polymerase I transcription factor UBF is a sequence-tolerant HMG-box protein that can recognize structured nucleic acids.. Nucleic Acids Res.

[37] Tseng H (2006). Cell-type-specific regulation of RNA polymerase I transcription: a new frontier.. BioEssays.

[38] Brown DD, Dawid IB (1968). Specific gene amplification in oocytes. Oocyte nuclei contain extrachromosomal replicas of the genes for ribosomal RNA.. Science.

[39] Perkowska E, Macgregor HC, Birnstiel ML (1968). Gene amplification in the oocyte nucleus of mutant and wild-type Xenopus laevis.. Nature.

[40] Young DW, Hassan MQ, Pratap J, Galindo M, Zaidi SK (2007). Mitotic occupancy and lineage-specific transcriptional control of rRNA genes by Runx2.. Nature.

[41] Buttgereit D, Pflugfelder G, Grummt I (1985). Growth-dependent regulation of rRNA synthesis is mediated by a transcription initiation factor (TIF-IA).. Nucleic Acids Res.

[42] Bodem J, Dobreva G, Hoffmann-Rohrer U, Iben S, Zentgraf H (2000). TIF-IA, the factor mediating growth-dependent control of ribosomal RNA synthesis, is the mammalian homolog of yeast Rrn3p.. EMBO Rep.

[43] Yuan X, Zhao J, Zentgraf H, Hoffmann-Rohrer U, Grummt I (2002). Multiple interactions between RNA polymerase I, TIF-IA and TAF(I) subunits regulate preinitiation complex assembly at the ribosomal gene promoter.. EMBO Rep.

[44] Yuan X, Zhou Y, Casanova E, Chai M, Kiss E (2005). Genetic inactivation of the transcription factor TIF-IA leads to nucleolar disruption, cell cycle arrest, and p53-mediated apoptosis.. Mol Cell.

[45] Russell J, Zomerdijk JC (2005). RNA-polymerase-I-directed rDNA transcription, life and works.. Trends Biochem Sci.

[46] de Capoa A, Marlekaj P, Baldini A, Rocchi M, Archidiacono N (1985). Cytologic demonstration of differential activity of rRNA gene clusters in different human tissues.. Hum Genet.

[47] de Capoa A, Marlekaj P, Baldini A, Felli MP, Rocchi M (1986). Growth hormone-induced regulation of rRNA gene activity in human cultured cells.. Horm Metab Res.

[48] de Capoa A, Marlekaj P, Baldini A, Archidiacono N, Rocchi M (1985). The transcriptional activity of individual ribosomal DNA gene clusters is modulated by serum concentration.. J Cell Sci.

[49] Haaf T, Hayman DL, Schmid M (1991). Quantitative determination of rDNA transcription units in vertebrate cells.. Exp Cell Res.

[50] Corpet F (1988). Multiple sequence alignment with hierarchical clustering.. Nucleic Acids Res.

[51] Storey JD, Tibshirani R (2003). Statistical significance for genome-wide studies.. Proc Natl Acad Sci U S A.

